# Supplementary material for: Rapid and sensitive on‐site genetic diagnostics of pest fruit flies using CRISPR‐Cas12a
Source: Pest Manag Sci. 2022 Sep 22;79(1):68–75. doi: 10.1002/ps.7173 (PMC10086973; doi:10.1002/ps.7173)
Supplement: Supplementary file 1 — Figure S1. gRNA testing for B. zonata. Figure S2. Sensitivity testing for B. zonata‐specific identification. Figure S3. Variable region 1 multiple sequence alignment of various pest species. Figure S4. In‐depth analysis of the Bz1 VR gRNA target sites between various pest species. Figure S4. In‐depth analysis of the Bz1 VR gRNA target sites between various pest species. Table S1. DNA sequences Table S2. Reagent costs [file PS-79-68-s001.docx]

Rapid and sensitive on-site genetic diagnostics of pest fruit flies using CRISPR-Cas12a

Dan Mark Alon^1,2^* , Tamir Partosh^1^, David Burstein^2^ and Gur Pines^1^*

^1^ Department of Entomology, Agricultural Research Organization - the Volcani Center, 68 HaMaccabim Rd, Rishon LeZion 7505101, Israel

^2^ The Shmunis School of Biomedicine and Cancer Research, Faculty of Life Science, Tel   Aviv University, Tel Aviv 69978, Israel

* Corresponding authors: [gurpines@volcani.agri.gov.il](mailto:gurpines@volcani.agri.gov.il)

[alondanm@gmail.com](mailto:alondanm@gmail.com)

Supplementary material

Contents

[**Figure S1- gRNA testing for *B. zonata*** 3](#_Toc112093900)

[**Figure S2 - Sensitivity testing for *B. Zonata-specific* identification** 4](#_Toc112093901)

[**Figure S3 – Variable region 1 multiple sequence alignment of various pest species** 5](#_Toc112093902)

[**Figure S4 – In-depth analysis of the Bz1 VR gRNA target sites between various pest species** 6](#_Toc112093903)

[**Table S1 -DNA Sequences** 7](#_Toc112093904)

[**Table S2 - Reagent Costs** 9](#_Toc112093905)

## **Figure S1- gRNA testing for *B. zonata***

**
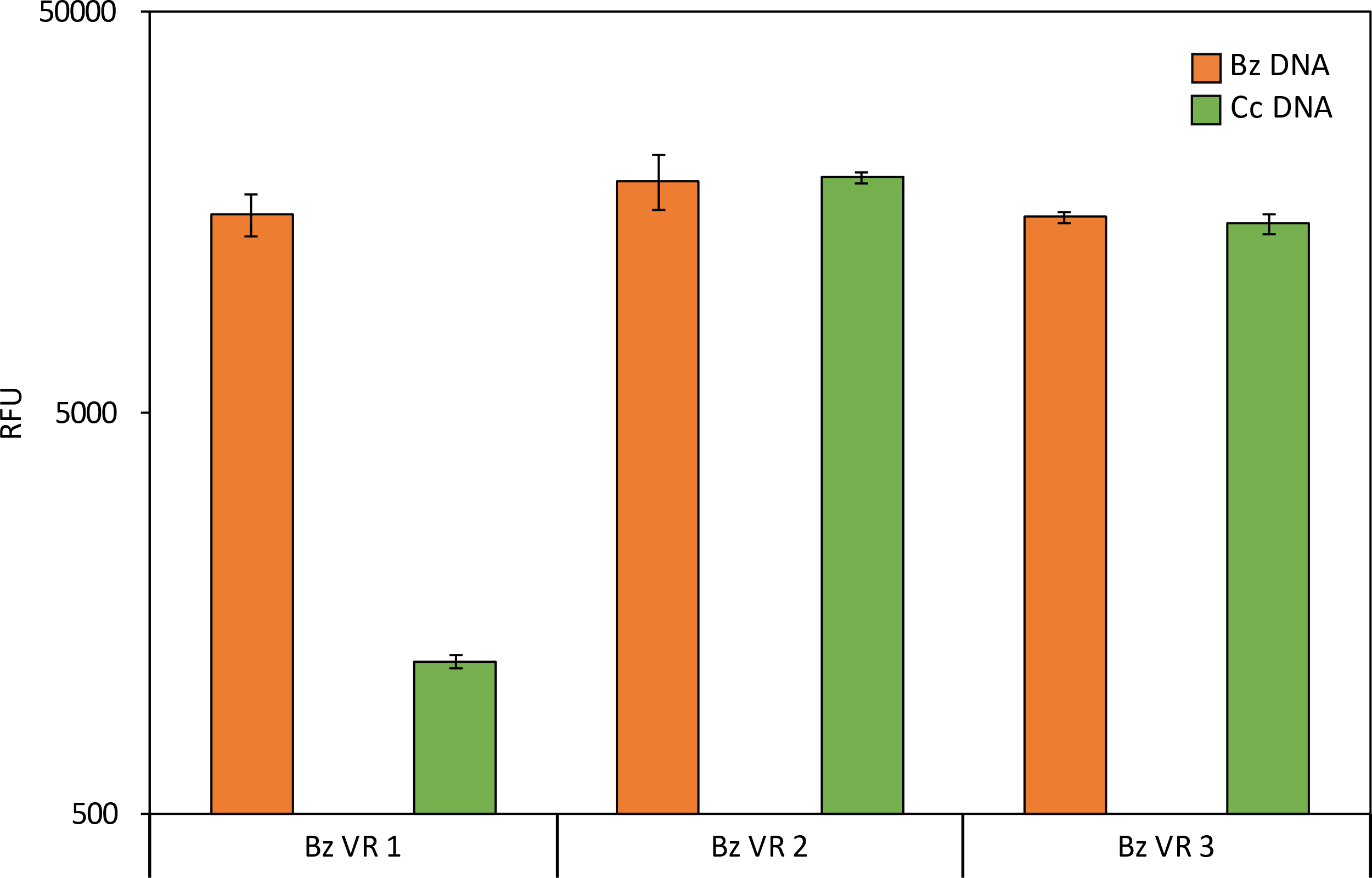
**

Testing of three different gRNAs based on CRISPOR analysis of the *B. zonata* variable region. *B. zonata* larvae were used, and amplification was performed using PCR with B. *Zonata-specific* primers (Table S1). Negative control was performed on *C. capitata* larval DNA, amplified with the primers described above. All experiments were performed with three biological repeats and three technical repeats.

## **Figure S2 - Sensitivity testing for *B. Zonata-specific* identification**

**
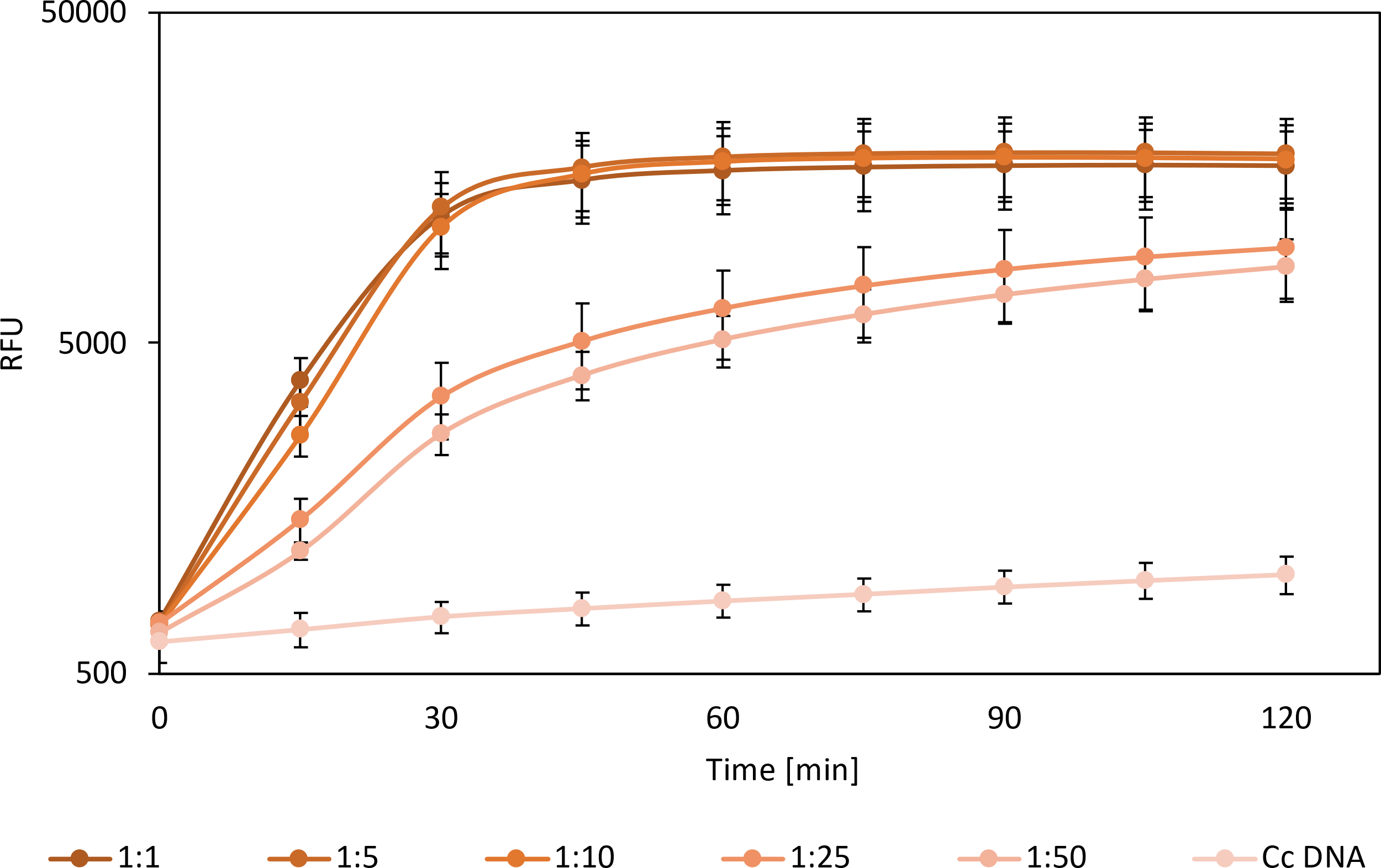
**

Detection sensitivity of Cas12a-Bz1 gRNA in varying ratios of pooled larvae. Samples containing a single larva of *B. zonata* with increasing amounts of *D. melanogaster* larvae were prepared. DNA was then extracted using Chelex 100, RPA amplified using *B. zonata* specific primers (Table S1), and diagnosed using Cas12a-Bz1 gRNA complexes. As a negative control, *C. capitata* DNA was used. All experiments were performed with three biological repeats and three technical repeats.

## **Figure S3 – Variable region 1 multiple sequence alignment of various pest species**


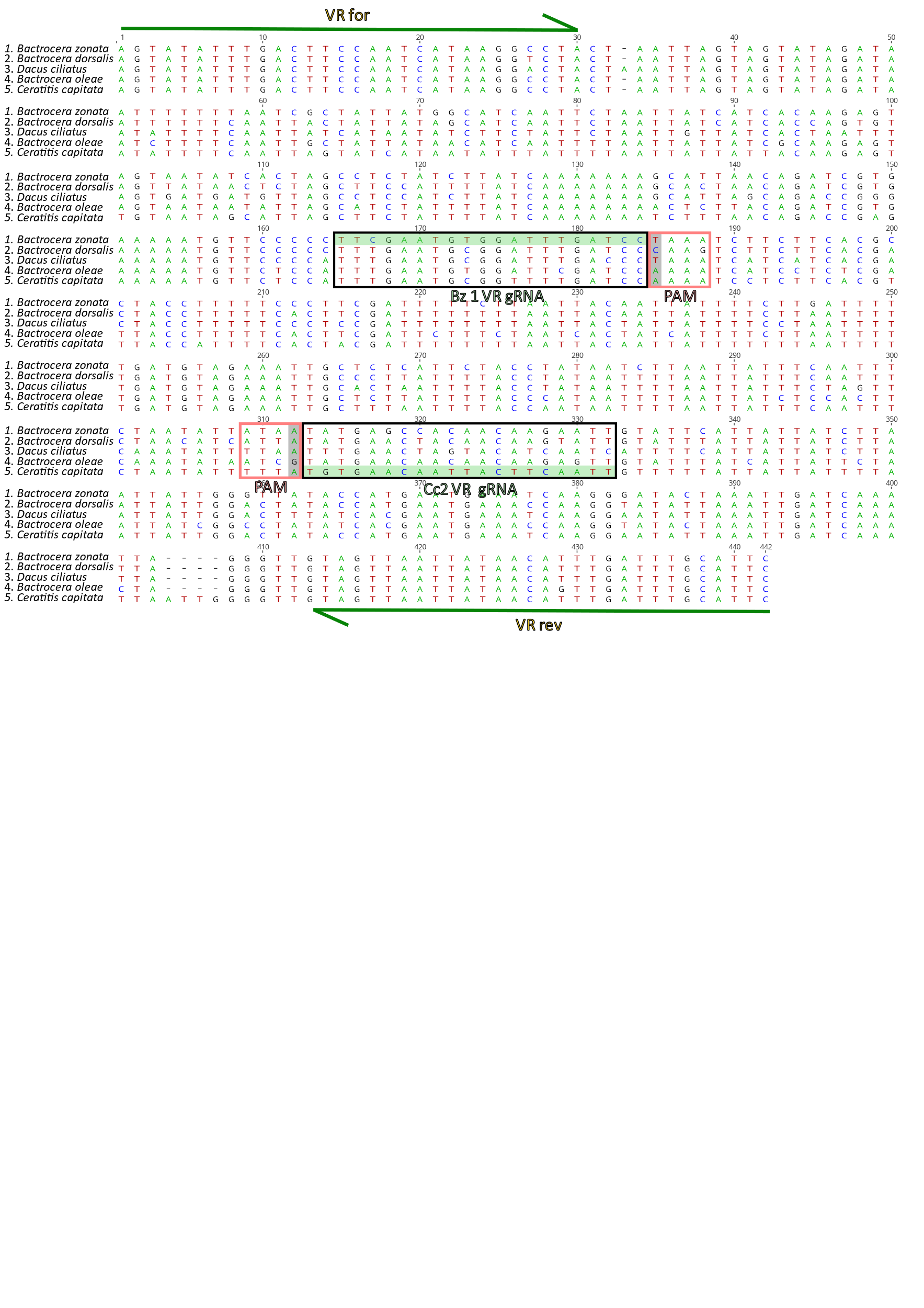


A comparison of the mitochondrial variable region 1 (VR1) sequence of different fruit fly pests’ species. Green arrows indicate universal primers binding sites, gRNA target sites are marked within the black boxes and PAM recognition sequences are marked within a red box.

## **Figure S4 – In-depth analysis of the Bz1 VR gRNA target sites between various pest species**


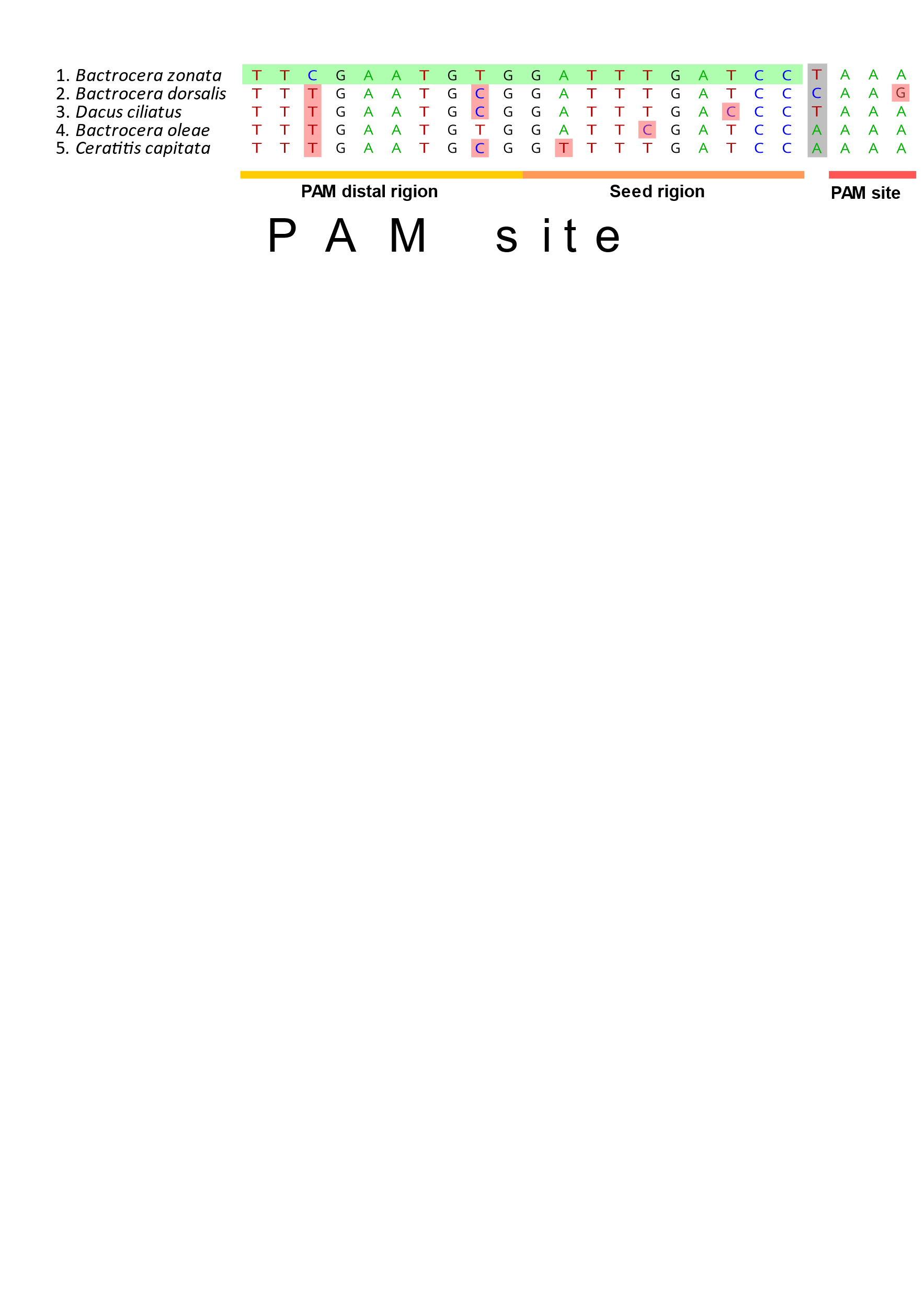


A multiple sequence alignment of the Bz1 VR gRNA target sequences of different fruit fly pests’ species. Bz1 VR gRNA is highlighted in green, and variations from the original gRNA sequence are highlighted in red.

## **Table S1 -DNA Sequences**

| **Name** | **Sequence** | **Description** |
| --- | --- | --- |
| Cc2 VR | TGTGAACAATTACTTCAATTGTT | gRNA |
| Bz1 VR | GGATCAAATCCACATTCGAAGGG | gRNA |
| FAM sensor | /56-FAM/TTATTATT/3BHQ_1/ | ssDNA probe |
| VR Bz for | AGAGTAGTAATATCACTAGCCTCTATCTTAT | *Bactrocera zonata* specific amplification primer |
| VR Bz rev | CTACAACCCTAATTTGATCAATTTAGTATC | *Bactrocera zonata* specific amplification primer |
| VR for | AGTATATTTGACTTCCAATCATAAGGCCTAC | Universal amplification primer |
| VR rev | GAATGCAAATCAAATGTTATAATTAACTAC | Universal amplification primer |
| *B. zonata* variable region 1 | AGTATATTTGACTTCCAATCATAAGGCCTACTAATTAGTAGTATAGATAATTTTTTTAATCGCTATTATGGCATCAATTCTAATTATCATCACAAGAGTAGTAATATCACTAGCCTCTATCTTATCAAAAAAAGCATTAACAGATCGTGAAAAATGTTCCCCCTTCGAATGTGGATTTGATCCTAAATCTTCTTCACGCCTACCTTTTTCCCTTCGATTTTTCTTAATTACAATTATTTTCTTGATTTTTGATGTAGAAATTGCTCTCATTCTACCTATAATCTTAATTATTTCAATTTCTAATATTATAATATGAGCCACAACAAGAATTGTATTCATTATTATCTTAATTATTGGGTTATACCATGAATGAAATCAAGGGATACTAAATTGATCAAATTAGGGTTGTAGTTAATTATAACATTTGATTTGCATTCAAAAAGTATTGA | Genomic sequence |
| *C. capitata* variable region 1 | AGTATATTTGACTTCCAATCATAAGGCCTACTAATTAGTAGTATAGATAATATTTTCAATTAGTATCATAATATTTATTTTAATTATTATTACAAGAGTTGTAATAGCATTAGCTTCTATTTTATCAAAAAAATCTTTAACAGACCGAGAAAAATGTTCTCCATTTGAATGCGGTTTTGATCCAAAATCCTCTTCACGTTTACCATTTTCACTACGATTTTTTTTAATTACAATTATTTTTTTAATTTTTGATGTAGAAATTGCTTTAATTTTACCAATAATTTTAATTATTTCAATTTCTAATATTTTTATGTGAACAATTACTTCAATTGTTTTTATTATTATTTTAATTATTGGACTATACCATGAATGAAATCAAGGAATATTAAATTGATCAAATTAATTGGGGTTGTAGTTAATTATAACATTTGATTTGCATTCAAAAAGTATTGAATTTCAATCTACCTTATTATTTTCAAGAATATGAAGCGATTTATTGCAATTAGTTTCGACCTAATCTTAGGTTT | Genomic sequence |
| Concatenated variable regions | AGTATATTTGACTTCCAATCATAAGGCCTACTAATTAGTAGTATAGATAATATTTTCAATTAGTATCATAATATTTATTTTAATTATTATTACAAGAGTTGTAATAGCATTAGCTTCTATTTTATCAAAAAAATCTTTAACAGACCGAGAAAAATGTTCTCCATTTGAATGCGGTTTTGATCCAAAATCCTCTTCACGTTTACCATTTTCACTACGATTTTTTTTAATTACAATTATTTTTTTAATTTTTGATGTAGAAATTGCTTTAATTTTACCAATAATTTTAATTATTTCAATTTCTAATATTTTTATGTGAACAATTACTTCAATTGTTTTTATTATTATTTTAATTATTGGACTATACCATGAATGAAATCAAGGAATATTAAATTGATCAAATTAATTGGGGTTGTAGTTAATTATAACATTTGATTTGCATTCAAAAAGTATTGAATTTCAATCTACCTTATTATTTTCAAGAATATGAAGCGATTTATTGCAATTAGTTTCGACCTAATCTTAGGTTTccccccccccAGTATATTTGACTTCCAATCATAAGGCCTACTAATTAGTAGTATAGATAATTTTTTTAATCGCTATTATGGCATCAATTCTAATTATCATCACAAGAGTAGTAATATCACTAGCCTCTATCTTATCAAAAAAAGCATTAACAGATCGTGAAAAATGTTCCCCCTTCGAATGTGGATTTGATCCTAAATCTTCTTCACGCCTACCTTTTTCCCTTCGATTTTTCTTAATTACAATTATTTTCTTGATTTTTGATGTAGAAATTGCTCTCATTCTACCTATAATCTTAATTATTTCAATTTCTAATATTATAATATGAGCCACAACAAGAATTGTATTCATTATTATCTTAATTATTGGGTTATACCATGAATGAAATCAAGGGATACTAAATTGATCAAATTAGGGTTGTAGTTAATTATAACATTTGATTTGCATTCAAAAAGTATTGA |  |

## **Table S2 - Reagent Costs**

|  | **Reactions** | **Cost (USD)** |
| --- | --- | --- |
| [TwistAmp® Basic](https://www.twistdx.co.uk/product/twistamp-basic/) | 480 | 253 |
| [Lb cas12a NEB](https://international.neb.com/products/m0653-engen-lba-cas12a-cpf1#Product%20Information) | 426 | 400 |
| [gRNA IDT](https://eu.idtdna.com/pages/products/crispr-genome-editing/alt-r-crispr-cpf1-genome-editing) (10nmol) | 1600 | 140 |
| Cost per reaction ($): | | 1.6 |
